# Weaning stress induced alterations to the hypothalamic‐pituitary‐thyroid and ‐adrenal axes in swine

**DOI:** 10.14814/phy2.70984

**Published:** 2026-06-26

**Authors:** Isabel B. Walpole, Alyssa A. Smith, Kaylyn G. Rudy, Dayeon Jeon, J. Scott Radcliffe, J. Alex Pasternak

**Affiliations:** ^1^ Department of Animal Sciences Purdue University West Lafayette Indiana USA; ^2^ Department of Animal and Food Sciences University of Kentucky Lexington Kentucky USA

**Keywords:** allostasis, non‐thyroidal illness syndrome, stress, swine, thyroid

## Abstract

Combined modulation of the hypothalamic–pituitary–thyroid (HPT) and hypothalamic–pituitary–adrenal (HPA) axes is known to play a critical role in maintaining physiological stability in response to stress. In this study, stress induced modulation of these axes was examined using 20 pigs/treatment either weaned (WN), weaned with feed/water restriction 9 h (FR), transported 9 h (TRANS), or remaining with sow (SOS) and euthanized at 32 or 56 h post‐weaning. Serum cortisol, thyroxine (T4), and triiodothyronine (T3) were measured before weaning and after 24, 32, and 56 h with no difference in cortisol concentrations among treatments at any timepoint. However, both T3 and T4 decreased post‐weaning in FR, TRANS, and WN compared to SOS. Expression of 23 genes of interest was evaluated across the hypothalamus, pituitary, thyroid, and adrenal glands of TRANS and SOS pigs at 56 h to understand the underlying mechanism. *TSHB*, *DIO3*, and *POMC* expression was decreased in the pituitary while *DIO3* in the thyroid was increased, with upregulation in *MC2R* also observed. Collectively, these results suggest acute stressors in swine can induce type I allostasis within the HPT axis and modulate sensitivity within the HPA.

## 1 INTRODUCTION

In many mammalian species, stress has been connected to endocrine modulation, but the physiological mechanism behind this connection has not been fully elucidated. The most well understood response to stress is within the hypothalamic–pituitary–adrenal (HPA) axis, which is responsible for production of cortisol, which is well‐documented in response to both physical (James et al., 2023) and psychological stress (Burke et al., 2005). While beneficial in the short term, excessive or chronic elevations in cortisol are associated with an increased risk for digestive, heart, metabolic, and immune disorders (Dhama et al., 2019). In swine, the rapid social, environmental, and nutritional transition at weaning creates a significant stressor that results in an increase in cortisol (Kojima et al., 2008; Sutherland, Bryer, et al., 2009; Sutherland, Krebs, et al., 2009). This elevation in cortisol may be exacerbated by additional factors such as handling or transport from one facility to another, which commonly coincides with weaning. This weaning‐related stress and cortisol elevation is often correlated with decreased feed intake and growth, which is an undesirable response in swine production (Papatsiros et al., 2024).

Stress induction of the HPA axis begins with the release of corticotropin‐releasing hormone (CRH) from the hypothalamus, which binds to CRH receptor 1 (CRHR1) and 2 (CRHR2) in the pituitary. CRHR1 activation stimulates production of proopiomelanocortin (POMC), the precursor to adrenocorticotropic hormone (ACTH; Harno et al., 2018; Hillhouse & Grammatopoulos, 2006) while CRHR2 modulates the stress response (Kishimoto et al., 2000). ACTH, in turn, elicits an adrenal response via the melanocortin 2 receptor (MC2R), stimulating production of glucocorticoids such as cortisol (Lefebvre et al., 2016). This steroidogenesis pathway begins with cholesterol transport from the outer to the inner mitochondrial membrane, which is mediated by the steroidogenic acute regulatory protein (STAR; Miller, 2007). Cholesterol is then converted to pregnenolone by the cytochrome P450 family 11 subfamily A member 1 (CYP11A1; Chien et al., 2017), which, following a series of intermediate enzymatic reactions, can be converted into either aldosterone or cortisol by members of the cytochrome P450 family 11 subfamily B (CYP11B; Robic et al., 2014). In addition to activation of the HPA axis, stress also results in adrenal production of catecholamines including norepinephrine and epinephrine. These neurotransmitters are derived from tyrosine through the sequential action of enzymes including tyrosine hydroxylase (TH), dopa decarboxylase (DDC), dopamine β‐hydroxylase (DBH), and phenylethanolamine N‐methyltransferase (PNMT).

In contrast to the HPA, the hypothalamic–pituitary–thyroid (HPT) axis is typically under strict homeostatic control, but is known to exhibit allostatic regulation in response to various environmental and physiological stressors. In type 1 allostasis, the physiological set point within the HPT axis is reduced in response to starvation, exhaustion, or disease (Chatzitomaris et al., 2017). In contrast, type 2 allostasis is characterized by an increase in hormone abundance in response to obesity or in an effort to adapt to cold (Chatzitomaris et al., 2017). In non‐thyroidal illness syndrome (NTIS), type 1 allostatic regulation results in decreased circulating thyroid hormone levels, without the typical compensatory increase in TSH (Chatzitomaris et al., 2017). Swine have been shown to exhibit an NTIS‐like response to pathogenic stress including infection with porcine circovirus (Harding et al., 2022), infection with porcine reproductive and respiratory syndrome virus (PRRSV; Pasternak et al., 2020), and a polymicrobial disease challenge (Pasternak et al., 2021). Independent of weaning, transportation has been shown to have similar effects on T3 concentrations, which decrease after a 1 h transport (Yoshioka et al., 2004). Furthermore, in humans, NTIS has been observed during critical illness, starvation, and other stressful events like exhaustive exercise (Chatzitomaris et al., 2017) with acute exercise stress shown to have a similar effect in horses (Ferlazzo et al., 2014).

Much like the HPA axis, activity within the HPT axis is initiated by hypothalamic release of thyrotropin‐releasing hormone (TRH) which elicits a pituitary response via the TRH receptor (TRHR). This stimulates the release of thyroid‐stimulating hormone (TSH), a glycoprotein hormone comprised of a common alpha and unique beta subunit (TSHB) which promotes productive activity within the thyroid through its corresponding receptor (TSHR). Within the thyroid follicle, tyrosine residues in the precursor thyroglobulin (TG) are iodinated, fused, and cleaved to produce thyroxine (T4) or triiodothyronine (T3). Following release, these amine hormones are transported across cell membranes by two primary solute carriers: solute carrier family 16 member 2 (SLC16A2) and solute carrier family 16 member 10 (SLC16A10; Abe et al., 2012; Arjona et al., 2011). Within the cytoplasm, T4 can be converted to the more bioactive T3 by iodothyronine deiodinase 2 (DIO2) or rendered inert in the form of reverse triiodothyronine (rT3), which is produced by iodothyronine deiodinase 3 (DIO3). Canonically, the cellular response to thyroid hormone is primarily mediated by a pair of nuclear receptors, thyroid hormone receptor α (THRA) and thyroid hormone receptor β (THRB).

Stress responses within the HPT and HPA axes may not occur independently, as cortisol is known to regulate thyroid hormone production by decreasing TSH and TRH release (Cintra et al., 1990; Samuels et al., 1994). Similarly, thyroid hormones cause an increase in *POMC* gene expression in the pituitary (Shi et al., 1994), while TSH increases ACTH release (Prévide et al., 2021). The net effect of this regulation is a decrease in thyroid hormone in response to elevated cortisol during stress. Given these established interconnections, it may be prudent to view these axes in conjunction in order to better understand endocrine disruption in response to postnatal stress. In the present study, we hypothesized that the stress associated with porcine weaning and transportation would elevate circulating cortisol and decrease circulating T4 and T3, and that this response would be proportional to the severity of the stressor. We further hypothesized that the combination of weaning and transport would alter transcriptional activity within the hypothalamic–pituitary–thyroid and adrenal (HPTA) axis in a manner consistent with type I allostasis.

## 2 MATERIALS AND METHODS

### 2.1 Animal model

All animal work was carried out at the Purdue University Animal Science Research and Education Center in compliance with Institutional Care and Use Committee regulations under an approved protocol (IACUC #0123002344). Animal care and use standards were based upon the Guide for the Care and Use of Agricultural Animals in Research and Teaching (Tucker et al., 2020).

A total of *n* = 346 objectively healthy pigs, housed in standard lactation crates with their associated dam, were weighed 7 days prior to weaning and a subset of *n* = 40 gilts and *n* = 40 barrows with the lowest within litter *z*‐scores were selected for study (3.91 ± 0.39 kg). At weaning (20 ± 1.3 days), an equal number of barrows and gilts were assigned to 1 of 4 treatments including: (1) weaning directly into nursery pens with access to feed and water (WN), (2) weaning into pens without access to feed or water for 9 h (FR), (3) weaning and immediate transport for 9 h without access to feed or water (TRANS), and (4) not weaned, but transferred to nurse sows with 10 pigs/sow (stay on sow; SOS). Transportation was carried out in a straw‐bedded bumper pull livestock trailer loaded at 0.066 m^2^/pig density with pigs transported on 3 loops of a 3‐h route with a mix of two and four lane highways. In order to replicate industry standard weaning and transport practices, no additional interventions to alleviate pain or discomfort associated with weaning feed restriction or transport were utilized. Outside temperature and humidity during transport were 22°C–29°C and 67%–93%, respectively. All FR and TRANS pigs had access to ad libitum feed and water after the initial 9‐h period. Serum was collected 24 h prior to weaning (prewean), and at 24 and 48 h post‐weaning, and during euthanasia at 32 or 56 h post‐weaning. Serum at 32 h post‐weaning was collected only from animals euthanized at that time, while those at 48 and 56 h post‐weaning were only collected from pigs euthanized at 56 h.

### 2.2 Sample collection

At 32 h post‐weaning, *n* = 5 barrows and *n* = 5 gilts per treatment group were humanely euthanized via carbon dioxide followed by exsanguination as described in the AVMA guidelines (Leary, 2020). The remaining *n* = 5 barrows and *n* = 5 gilts per treatment group were euthanized at 56 h post‐weaning. Thyroid (ROID), diencephalon including the hypothalamus (HYP), pituitary (PIT), and adrenal glands (ADR) were collected, flash frozen in liquid nitrogen, and subsequently stored at −80°C until further analysis.

### 2.3 Endocrine assays

Total T3 and T4 concentrations in serum were measured in a total of *n* = 280 and *n* = 279 samples, using commercially available chemiluminescence immunoassays (MP Biomedical, Solon, USA, Cat# MP07M175 & MP07M275) as previously described (Smith et al., n.d.; Ison et al., 2023). Serum concentrations were measured preweaning, and at 24, 32, 48, and 56 h post‐weaning. Assays were carried out on *n* = 80 samples from preweaning and 24 h post‐weaning, and *n* = 40 samples from the remaining three timepoints. Average inter‐ and intra‐assay coefficients of variance were determined to be 12.63% and 6.08% for T3 and 9.42% and 5.69% for T4, respectively. Total serum cortisol was similarly measured in a total of *n* = 239 samples using a commercially available chemiluminescence immunoassay (MP Biomedical, Cat# 07 M3675) as per the manufacturer's guidelines, as previously demonstrated in swine (Smith et al., 2025). However, based on the T3 and T4 results, the cortisol assay was not run on 48 h post‐weaning samples. Average inter‐ and intra‐assay coefficients of variance were determined to be 12.71% and 6.37% for cortisol.

### 2.4 Gene expression

Given the uniform endocrine response between WN, FR, and TRANS, the latter was selected for indepth transcriptomic analysis of the corresponding axis relative to the SOS control. Samples of ROID, HYP, PIT, and ADR at 56 h post‐weaning were ground into a fine powder under cryogenic conditions using a mortar and pestle. RNA was extracted using TRIzol (Thermofisher Scientific, Waltham, USA, Cat# 15–596‐018) and a previously described double precipitation procedure (Mulligan et al., 2022). DNA contamination was removed using the TURBO DNase kit (Thermofisher Scientific, Cat# AM2238) following the manufacturer's protocol with the addition of a recombinant RNAse inhibitor (20 units/reaction; Thermofisher Scientific, Cat# N8080119). RNA concentration and purity were measured using a Nanodrop ND‐1000 (Thermofisher Scientific), and integrity assessed through denaturing agarose gel electrophoresis (Kent‐Dennis et al., 2019). A total of 2 μg of purified RNA was used to synthesize cDNA using the High‐Capacity cDNA Reverse Transcription kit (Thermofisher Scientific, Cat# 4368814).

Gene‐specific primers were drawn from the literature or designed using current RefSeq mRNA sequences to cover all predicted transcript variants in the pig (Table 1). Where possible, primers were designed to span exon‐exon junctions as identified by the BLAST‐like alignment tool (BLAT), relative to the *Sus Scrofa* genome assembly Sscrofa 11.1. The post‐amplification melt curve for each primer pair indicated a single amplicon, and reaction efficiency was determined to fall between 95%–105% for each primer pair.

**TABLE 1:** Porcine specific primer sequences used for qPCR.

Function	Gene symbol	Gene ID	Tissue	Forward primer	Reverse primer	Target RefSeq or source
Enzyme	CYP11A1	403329	ADR	5′‐CGGCAACTTGGAATCTGTTT‐3′	5′‐GGCTCCTGACTTCTTCAGCA‐3′	NM_214427
	CYP11B	110260194	ADR	5′‐CAGTATGCCAACAAAGCCATC‐3′	5′‐CATGTTTGCGTGTGTCAGC‐3′	Smith et al. (2025)
	DBH	733609	ADR	5′‐CTGGATTCCCAGCAGGATTA‐3′	5′‐TGCCGTCCTCGATGAAGTA‐3′	Smith et al. (2025)
	DDC	396857	ADR	5′‐TCCTTTCTTCGTGGTGGCTA‐3′	5′‐GGAACTCAGGGCAGATGAAG‐3′	Smith et al. (2025)
	DIO2	414379	HYP, PIT, ROID, ADR	5′‐CTCGGTCATTCTCCTCAAGC‐3′	5′‐TCACCTGTTTGTAGGCATCG‐3′	Pasternak et al. (2020a)
	DIO3	414378	HYP, PIT, ROID, ADR	5′‐CCTATCTGCGTGTCTGACGA‐3′	5′‐GCCTGCTTGAAGAAATCCAG‐3′	Pasternak et al. (2020a)
	PNMT	100144479	ADR	5′‐CGATTGGAGTGTCTACAGCC‐3′	5′‐ACGTCGATGGGCAGGATC‐3′	Smith et al. (2025)
	STAR	396597	ADR	5′‐TGCTGAGTAAAGTGATCCCAGA‐3′	5′‐GCAGGATCTTGATCTTCTTGACA‐3′	Smith et al. (2025)
	TH	110259705	ADR	5′‐GGAGAACAAGGTCCTCTGGT‐3′	5′‐GGTACACCTGGTCCGAGAAG‐3′	Smith et al. (2025)
Ligand or Precursor	CRH	100127468	HYP	5′‐TGGATCTCACCTTCCACCTC‐3′	5′‐CCATCAGTTTCCTGTTGCTG‐3′	Smith et al. (2025)
	POMC	396863	PIT	5′‐GGAAGATGCCGAGATTGTGC‐3′	5′‐GCACGCCAGCAAGTTACTTT‐3′	Smith et al. (2025)
	TG	100156471	ROID	5′‐CAGTGGCTTCTTCGAGTGTG‐3′	5′‐CGTCACCTCTCCTCCTTTCA‐3′	Smith et al. (2025)
	TRH	100513309	HYP	5′‐CAGTGGCTTCTTCGAGTGTG‐3′	5′‐CGTCACCTCTCCTCCTTTCA‐3′	Smith et al. (2025)
	TSHB	397658	PIT	5′‐ATGACACGGGATTTCAATGG‐3′	5′‐GTGGGCATCCTGGTATTTCT‐3′	Smith et al. (2025)
Receptor	CRHR1	397426	PIT	5′‐ATGAGAAGTGCTGGTTTGGC‐3′	5′‐TGCGGACGATGTTGAAAAGG‐3′	Smith et al. (2025)
	CRHR2	100240719	PIT	5′‐CCTCATCGCCACCTTTATCC‐3′	5′‐CACCAGACCTCGTTGCTCT‐3′	Smith et al. (2025)
	MC2R	100739231	ADR	5′‐CCATTTCTGACATGCTGGGC‐3′	5′‐GGAGTCCACTACGTCGTCAG‐3′	Smith et al. (2025)
	THRA	397387	HYP, PIT, ROID, ADR	5′‐GAGGAGAACAGTGCCAGGTC‐3′	5′‐CGACACACTGCTCGTCTTTG‐3′	Pasternak et al. (2020a)
	THRB	396776	HYP, PIT, ROID, ADR	5′‐AAGGCTGCAAGGGTTTCTTT‐3′	5′‐TGGCACTGATTTCTGGTGAC‐3′	Pasternak et al. (2020a)
	TRHR	100415777	PIT	5′‐AGCCCAGTTTCTCTGCACAT‐3′	5′‐GTAGCCACAGGACACCACAAT‐3′	Smith et al. (2025)
	TSHR	397560	ROID	5′‐GACCAACCTTGCTGGATGTC‐3′	5′‐CAGGCTCCGAATACTGCTCT‐3′	NM_214297
Solute Carrier	SLC16A2	100513770	HYP, PIT, ROID, ADR	5′‐CACCCATTGCAGGGTTACTC‐3′	5′‐TATGGAGCCAAGGGATGAAA‐3′	Pasternak et al. (2020a)
	SLC16A10	100513513	HYP, PIT, ROID, ADR	5′‐AGTGGAGTTCCAAGCAGCAT‐3′	5′‐AGCCCAAACGATCAGTGAAT‐3′	Pasternak et al. (2020a)
Reference	B2M	397033	PIT, ROID	5′‐CTGGACGTGGGCTATAAATG‐3′	5′‐GGCGTGAGTAAACCTGAACC‐3′	Pasternak et al. (2020b)
	GAPDH	396823	ADR	5′‐CCTGGAGAAACCTGCAAAAT‐3′	5′‐TTGACGAAGTGGTCGTTGAG‐3′	Käser et al. (2015)
	RPL19	396989	HYP, PIT	5′‐AACTCCCGTCAGCAGATCC‐3′	5′‐AGTACCCTTCCGCTTACCG‐3′	Pasternak et al. (2015)
	STX5	100628048	HYP, ADR	5′‐TGCAGAGTCGTCAGAATGGA‐3′	5′‐CCAGGATTGTCAGCTTCTCC‐3′	Pasternak et al. (2020a)
	YWHAZ	780440	HYP, ROID, ADR	5′‐TGATGATAAGAAAGGGATTGTGG‐3′	5′‐GTTCAGCAATGGCTTCATCA‐3′	Pasternak et al. (2020a)

A total of 5 reference genes and 23 genes of interest that relate to ROID and ADR regulation, including enzymes, ligands, precursors, receptors, and transporters were evaluated (Table 1). qPCR was carried out in duplicate on 20 ng cDNA using SsoAdvanced Universal SYBR Green Supermix (BioRad, Hercules, USA, Cat# 1725275) and a CFX qPCR system (BioRad). The stability of each reference gene was assessed, and the two or three most stable genes within each tissue were used to calculate a geometric mean to normalize the expression of the genes of interest. Reference genes *RPL19*, *YWHAZ*, and *STX5* were used in the HYP, *RPL19*, and *B2M* used in the PIT, *B2M*, and *YWHAZ* used in the ROID, and *YWHAZ*, *STX5*, and *GAPDH* used in the ADR. Samples with a ΔCT greater than five standard deviations from the mean within a group for a given gene within each tissue were removed from analysis. Two samples were removed from PIT gene expression due to failure to extract sufficient RNA and a failure to consistently amplify reference genes. Additionally, one sample was removed from ROID gene expression due to a failure to consistently amplify reference genes.

### 2.5 Statistical analyses

Data analysis and statistical tests were carried out in R version 4.4.1 (R Core Team, 2024) with the nlme (Pinheiro et al., 2025) and emmeans packages (Lenth et al., 2025). Serum T4, T3, and cortisol concentrations were analyzed with a linear mixed effect model with treatment by time as fixed effects, animal as a repeat measure, and a Tukey correction for multiple testing applied. This data is presented as the estimated marginal mean values along with the corresponding 95% confidence intervals. Heteroskedastic gene expression data were analyzed using the nonparametric Wilcoxon signed rank test within gene and tissue and is presented in the form of fold changes relative to the mean expression within the SOS group using the 2^−ΔΔCT^ method relative to the average of the SOS group. All gene expression data is presented on a log_10_ axis, in the form of boxplots (median and quartiles) with individual data points to better illustrate the underlying distribution. Finally, to effectively visualize fold changes across the four tissues within the HPTA axis, a multi‐tissue heat map was generated as previously described (Smith et al., n.d.) using a custom R script based on the Grid Graphics Package (R Core Team, 2024). A *p* value of *p* ≤ 0.050 was used as the threshold for determining statistical significance and a *p* value of 0.05 < *p* ≤ 0.1 was used as the threshold for determining statistical tendency.

## 3 RESULTS

### 3.1 Serum T4, T3, and cortisol concentrations

In prewean samples (24 h prior to weaning), serum T4 concentrations did not differ among treatments (Table 2). At 24 h post‐weaning, SOS T4 concentrations were significantly higher than WN, FR, and TRANS treatments (*p* < 0.05). WN and TRANS did not differ in T4 concentrations, but FR was significantly lower than WN (*p* = 0.038) and tended to be lower than TRANS concentrations (*p* = 0.074). At 32 h, SOS was still higher than TRANS and FR (*p* < 0.05) but did not differ from WN concentrations. However, WN, FR, and TRANS were not significantly different at this timepoint. At 48 and 56 h, T4 in SOS was markedly higher than the other three treatments (*p* < 0.01) while WN, FR, and TRANS did not differ. There was a significant treatment by time interaction (*p* < 0.01) with SOS T4 concentrations increasing slightly over time and the other three groups decreasing in similar magnitudes.

**TABLE 2:** Sera thyroxine (T4), triiodothyronine (T3), and cortisol concentrations (nmol/L) prewean (24 h prior to weaning), or at 24, 32, 48, or 56 h after weaning in 20 day old pigs either weaned only (WN), weaned with 9 h feed and water restriction (FR), weaned and transported for 9 h (TRANS), or age‐matched, unweaned contemporaries (SOS).

Parameter	Treatment				*p* Values					
	SOS	WN	FR	TRANS	SOS vs. WN	SOS vs. FR	SOS vs. TRANS	WN vs. FR	WN vs. TRANS	FR vs. TRANS
T4, nmol/L										
Prewean	80.0 (72.6, 87.3)	79.0 (71.7, 86.4)	76.1 (68.8, 83.5)	76.9 (69.5, 84.2)	0.998	0.880	0.933	0.944	0.977	0.999
24 h	94.0^a^ (86.7, 101)	79.4^ab^ (71.9, 86.9)	64.9^b^ (57.6, 72.3)	77.8^b^ (70.5, 85.1)	0.034	<0.001	0.014	0.038	0.991	0.074
32 h	81.4^a^ (71.3, 91.5)	64.9^b^ (54.9, 75.0)	57.9^b^ (47.9, 68.0)	60.6^b^ (50.5, 70.6)	0.107	0.008	0.024	0.763	0.929	0.983
48 h	99.1^a^ (89.1, 109)	48.5^b^ (38.4, 52.0)	42.0^b^ (31.9, 52.0)	45.3^b^ (35.2, 55.4)	<0.001	<0.001	<0.001	0.797	0.971	0.965
56 h	90.4^a^ (80.4, 101)	42.6^b^ (32.5, 52.7)	42.0^b^ (31.9, 52.0)	55.2^b^ (45.2, 65.3)	<0.001	<0.001	<0.001	1.00	0.297	0.256
T3, nmol/L										
Prewean	1.77 (1.56, 1.98)	2.00 (1.80, 2.21)	1.99 (1.78, 2.20)	1.97 (1.76, 2.18)	0.398	0.449	0.522	1.00	0.997	0.999
24 h	1.96^a^ (1.75, 2.17)	1.42^b^ (1.22, 1.63)	1.20^b^ (0.99, 1.41)	1.42^b^ (1.21, 1.63)	0.003	<0.001	0.003	0.424	1.00	0.438
32 h	1.50^a^ (1.21, 1.78)	1.25^ab^ (0.97, 1.54)	0.91^b^ (0.63, 1.19)	0.74^b^ (0.46, 1.03)	0.629	0.024	0.002	0.328	0.062	0.839
48 h	1.54^a^ (1.25, 1.82)	0.63^b^ (0.35, 0.91)	0.40^b^ (0.12, 0.68)	0.68^b^ (0.40, 0.96)	<0.001	<0.001	<0.001	0.667	0.994	0.503
56 h	1.24^a^ (0.95, 1.52)	0.46^b^ (0.18, 0.75)	0.58^b^ (0.30, 0.87)	0.67^b^ (0.39, 0.96)	0.001	0.009	0.032	0.933	0.720	0.969
Cortisol, nmol/L										
Prewean	197 (159, 235)	207 (170, 244)	219 (180, 258)	176 (137, 215)	0.981	0.851	0.867	0.972	0.654	0.408
24 h	117 (78.6, 155)	101 (64.3, 139)	138 (99.0, 177)	149 (111, 187)	0.942	0.862	0.638	0.533	0.297	0.98
32 h	171 (117, 224)	121 (69.7, 172)	175 (118, 231)	179 (125, 233)	0.542	1.00	0.996	0.502	0.407	1.00
56 h	211^ab^ (158, 265)	138^a^ (84.4, 192)	228^ab^ (174, 281)	279^b^ (226, 333)	0.226	0.973	0.290	0.094	0.002	0.535

*Note*: Results are presented as emmean with 95% confidence intervals. ^a,b^Uncommon superscripts within a row denote significant differences at *p* ≤ 0.05 between treatments.

In prewean samples, serum T3 concentrations did not differ among treatments (Table 2). At 24 h, SOS serum T3 concentrations were significantly higher than all three other treatments (*p* < 0.01) while T3 concentrations did not statistically differ among the other three treatments. At 32 h, SOS T3 was still higher than the FR and TRANS treatments (*p* < 0.05), but WN T3 was no longer different from SOS or FR. Likewise, FR and TRANS were not statistically different from each other, but WN T3 tended to be higher than TRANS (*p* = 0.062). At 48 h and 56 h, T3 concentrations in the SOS pigs were higher than all other treatments (*p* < 0.05), and the other three treatments did not significantly differ from each other. There was an effect of treatment by time (*p* < 0.01) with SOS T3 concentrations slightly decreasing over time, but the other three treatments showed a sharper decline over the 56 h post‐weaning.

In prewean and 24 and 32 h post‐wean samples, there were no significant differences among treatments in serum cortisol concentrations (Table 2). At 56 h, WN had significantly lower (*p* < 0.01) and tended to have lower (*p* = 0.094) cortisol concentrations than TRANS and FR, respectively. The SOS treatment was not different from any treatment at 56 h in serum cortisol, and the TRANS and FR treatments were not different from each other. Overall, time had a significant effect on cortisol concentration with concentrations decreasing between prewean and 24 h and then increasing from 24 to 56 h (*p* < 0.01), but the interaction of treatment by time was only a tendency (*p* = 0.067).

### 3.2 Gene expression in the HPTA axis

#### 3.2.1 Hypothalamus

**FIGURE 1:**
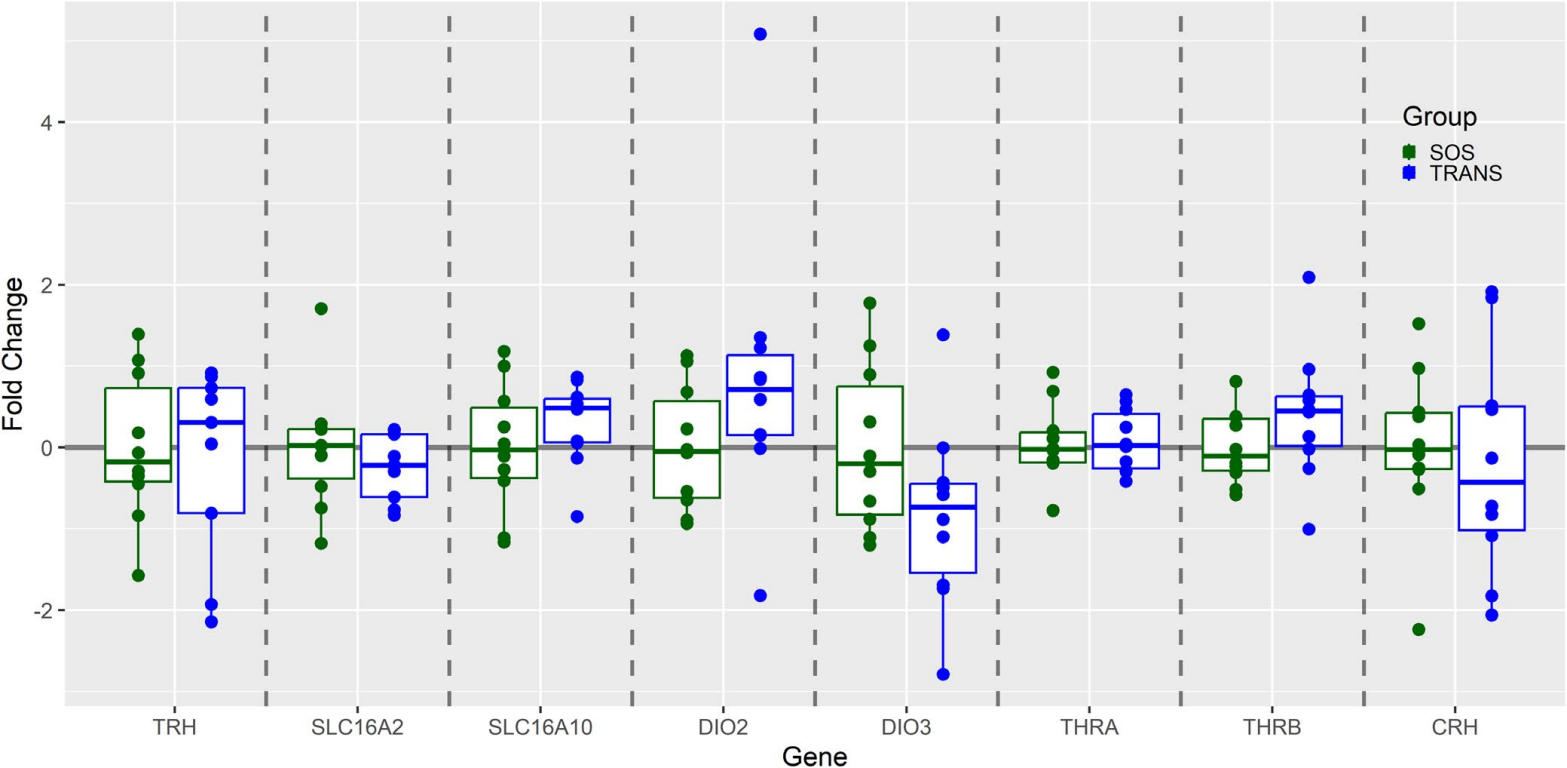
Expression of eight genes assessed in diencephalon including the hypothalamus (HYP) collected 56 h after combined weaning and 9 h transport (TRANS) of 20 days old piglets, compared to age matched, unweaned contemporaries (SOS). No statistically significant differences were observed in expression of any gene between the SOS and TRANS groups.

To evaluate the effects of weaning associated stress on the combined HPTA axis, expression of thyroid and adrenal regulating genes in the HYP, including *TRH*, *SLC16A2*, *SLC16A10*, *DIO2*, *DIO3*, *THRA*, *THRB*, and *CRH* was assessed. None of the selected genes were differentially expressed between SOS and TRANS treatments in the HYP (Figure 1).

#### 3.2.2 Pituitary

**FIGURE 2:**
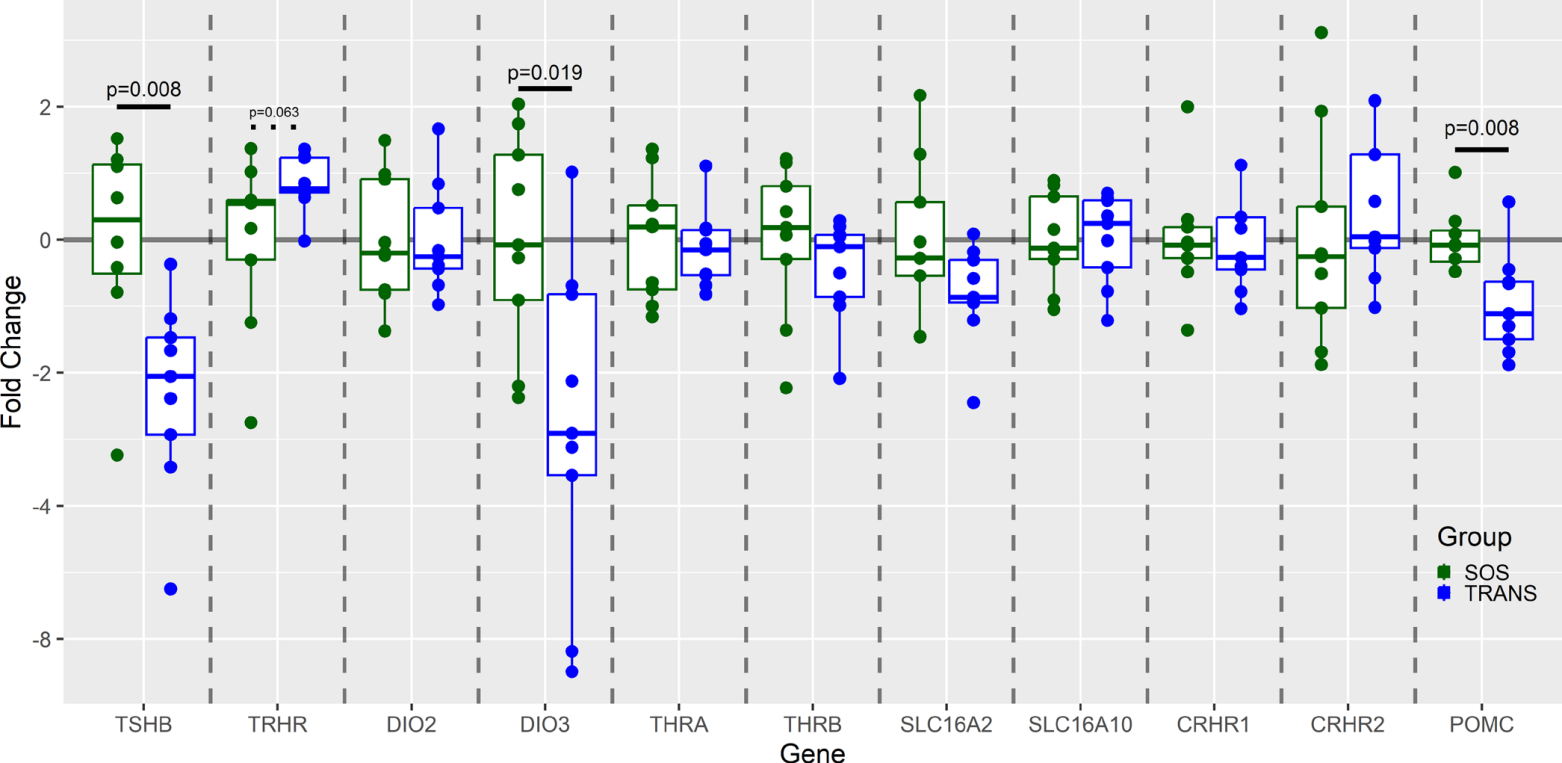
Expression of 11 genes assessed in pituitary (PIT) collected 56 h after combined weaning and 9 h transport (TRANS) of 20 days old piglets, compared to age matched, unweaned contemporaries (SOS).

Expression of receptors for hypothalamic ligands (*TRHR*, *CRHR1*, and *CRHR2*), transporters (*SLC16A2* and *SLC16A10*), enzymes (*DIO2* and *DIO3*), receptors regulating T3 and T4 tissue response (*THRA* and *THRB*), and ligands that regulate ROID (*TSHB*) and ADR (*POMC*) activity were assessed in the PIT (Figure 2). Expression of *TRHR* tended to be upregulated in the PIT (x̃ = −2.74 fold, *p* = 0.063) while TSH subunit *TSHB* was downregulated in TRANS pigs relative to SOS (x̃ = −2.04 fold, *p* < 0.01). Additionally, PIT expression of *DIO3* was also significantly downregulated in TRANS pigs (x̃ = 1.30 fold, *p* < 0.05), as well as the ACTH precursor, *POMC* (x̃ = −1.47 fold, *p* < 0.01). All other genes assessed in the PIT, including *DIO2*, *SLC16A2*, *SLC16A10*, *THRA*, *THRB*, *CRHR1*, and *CRHR2* did not differ in expression between TRANS and SOS pigs.

#### 3.2.3 Thyroid

**FIGURE 3:**
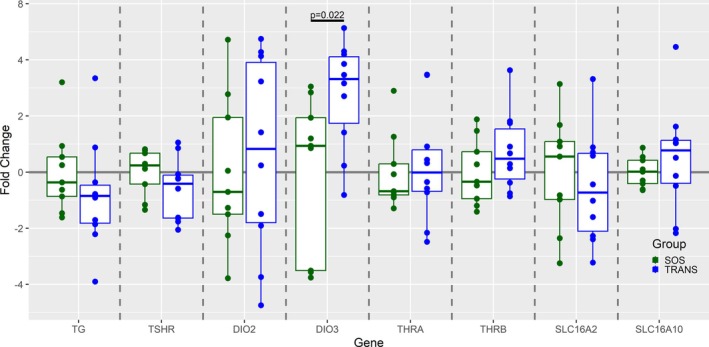
Expression of eight genes assessed in thyroid (ROID) collected 56 h after combined weaning and 9 h transport (TRANS) of 20 days old piglets, unweaned controls (SOS).

In the ROID, expression of genes related to production (*TG*), regulation (*TSHR*, *THRA*, and *THRB*), transport (*SLC16A2* and *SLC16A10*), and metabolism (*DIO2* and *DIO3*) of thyroid hormones was assessed (Figure 3). In contrast to observations in the PIT, *DIO3* was upregulated in the ROID of TRANS pigs relative to SOS (x̃ = 3.16 fold, *p* < 0.05). No other gene assessed in this tissue showed differential expression between TRANS and SOS pigs.

#### 3.2.4 Adrenal glands

**FIGURE 4:**
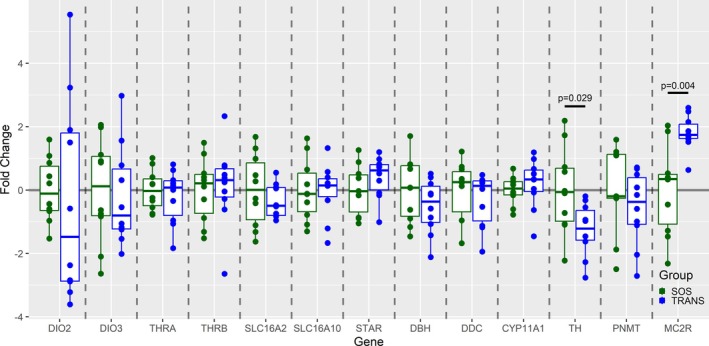
Expression of 13 genes assessed in adrenal gland (ADR) collected 56 h after combined weaning and 9 h transport (TRANS) of 20 days old piglets, compared to age matched, unweaned contemporaries (SOS).

In the ADR, expression of genes related to the regulation of steroidogenesis (*MC2R* and *STAR*), enzymes involved in steroidogenesis (*CYP11A1* and *CYP11B*), and enzymes involved in catecholamine synthesis (*TH*, *DDC*, *DBH*, and *PNMT*) were assessed, as well as transporters (*SLC16A2* and *SLC16A10*), receptors (*THRA* and *THRB*), and deiodinases (*DIO2* and *DIO3*) related to thyroid hormone action (Figure 4). ACTH receptor *MC2R* was upregulated in the ADR (x̃ = 3.16 fold, *p* < 0.01), and *TH* was downregulated in the ADR, of TRANS pigs compared to SOS pigs (x̃ = 3.16 fold, *p* < 0.05). No other gene in the ADR was differentially expressed between TRANS and SOS pigs.

#### 3.2.5 Overall

**FIGURE 5:**
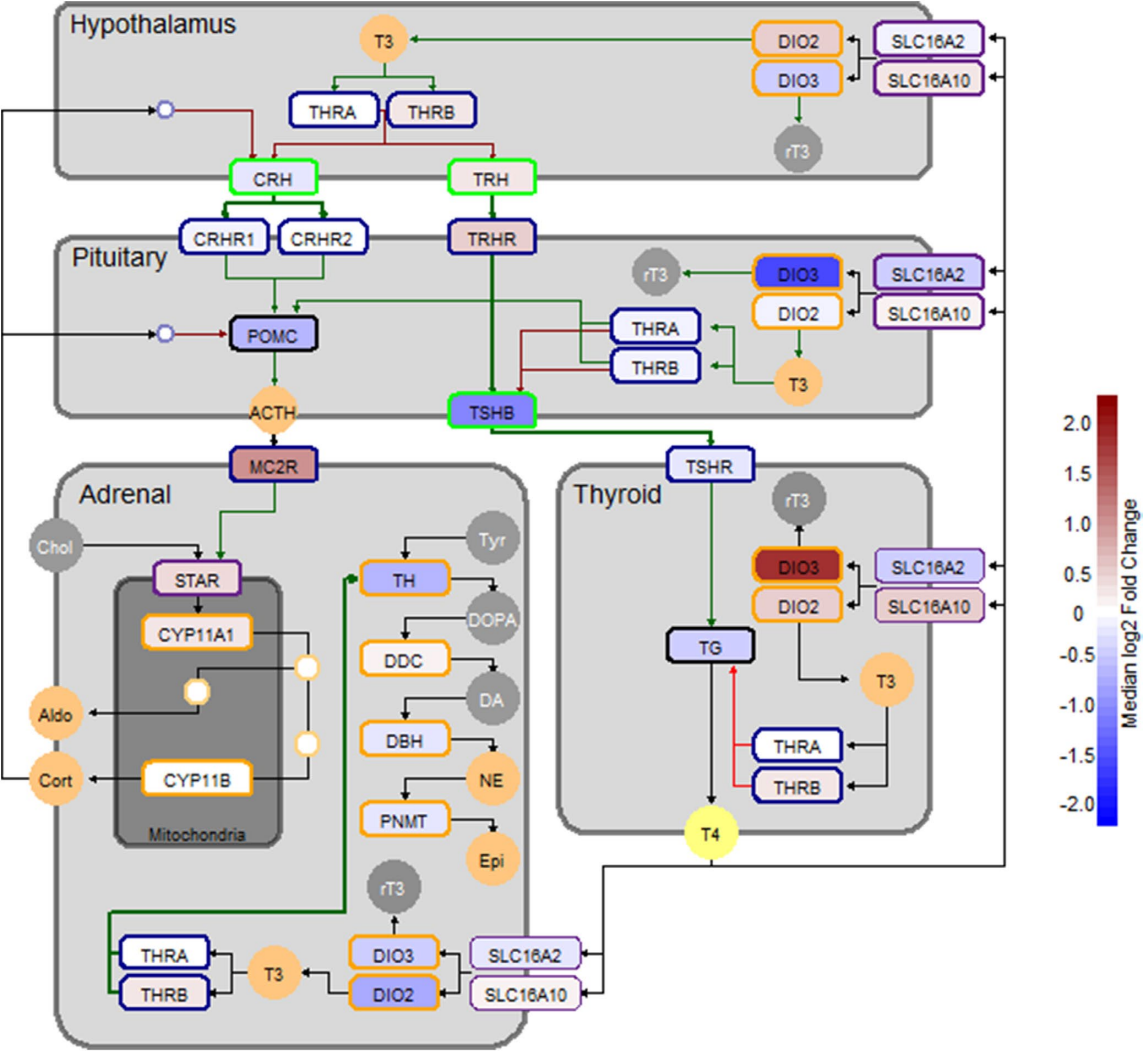
Hypothalamic–pituitary thyroid and adrenal (HPTA) axis gene expression fold changes in 20 days old piglets weaned and transported for 9 h compared to age matched, unweaned contemporaries (SOS). Fold changes were calculated relative to the average of the SOS group, with the direction and magnitude of each fold change indicated by fill color (labeled by gene). Genes encoding enzymes are outlined in orange, transports in purple, ligands in green and receptors in blue, with gray components indicating the tissue of origin. Substrates and metabolite hormones are represented by circles with abbreviations including Aldosterone (Aldo), cortisol (Cort), cholesterol (Chol), tyrosine (Tyr), dihydroxyphenylalanine (DOPA), dopamine (DA), norepinephrine (NE), and epinephrine (Epi).

To better understand the collective change in regulation of the entire HPTA axis, a multi‐tissue heat map was developed to visualize gene expression results across all four studied tissues (Figure 5). The map reiterates the reductions in expression of *TSHB* from the PIT, complemented by the increase in expression of *DIO3* in the ROID, but not in other tissues.

## 4 DISCUSSION

While stress is known to upregulate activity in the HPA axis, culminating in elevated circulating cortisol, the effect on other endocrine systems is not as well characterized. Prior work indicates that the fetal pig experiences endocrine modulation in both the HPA and HPT axes in response to pathogenic infection (Smith et al., n.d.). To determine if pigs near weaning age experience endocrine dysregulation in response to non‐pathogenic stressors, we first evaluated serum T3, T4, and cortisol following weaning, and coincident feed restriction and transport. Weaning is a common stressor in pigs' lives, with previous research demonstrating elevated cortisol and reduced growth after weaning (Sutherland, Krebs, et al., 2009). Additionally, weaning stress is typically composed of several coincident stressors including handling, diet change, feed restriction, and transportation. These coincident stressors allow for the study of varying degrees of stress including weaning alone, weaning and feed restriction, and weaning with transportation and feed restriction. In the present study, the use of these three weaning treatments allowed us to determine if short‐term modulation of the HPA and HPT axes differs by severity of stress, despite previous research indicating that long‐term growth is not impacted by transport or feed restriction at weaning (Walpole et al., 2025).

All weaned treatments experienced a decrease in T4 and T3 concentrations in serum, without the expected increase in serum cortisol concentrations. The lack of change in total serum cortisol after weaning and transportation observed in the present study was unexpected, but not unprecedented. Previous research has shown varying responses of serum cortisol to weaning, with a number of studies reporting no increase in serum cortisol (Averós et al., 2009; Carroll et al., 1998; Garcia et al., 2016), and others reporting an acute but transient increase in cortisol during the post‐weaning period (Dantzer et al., 1981; Kojima et al., 2008). In the present study, the first post‐weaning cortisol measurements were made at 24 and 32 h, and while this would be in line with peak cortisol in previous studies, it is possible it overlooked a more immediate response. Furthermore, all pigs, including SOS, were handled and restrained in the process of collecting serum, which in and of itself can elevate serum cortisol concentrations. One study found that for every minute that pigs were disturbed prior to blood collection, blood cortisol levels rose by 2.6 ng/mL (7.2 nmol/L; Garcia et al., 2016), such that sampling of group housed animals may artificially elevate cortisol. Additionally, to try and separate the effects of weaning, feed restriction, and transportation in the present study, all pigs regardless of treatment were separated from their birth sow and littermates and mixed into new groups. Handling and mixing combined may have elevated circulating cortisol in the SOS group, thereby masking any effect of weaning and transportation. Finally, the present study assessed total serum cortisol, which has been found to be a more sensitive measure of ADR activity, but assessment of salivary cortisol may be warranted in future studies due to its resistance to elevation from handling, and its correlation with biologically active free cortisol levels (Cook et al., 1996; Parrott et al., 1989). Sows' milk contains biologically relevant concentrations of cortisol which may be transferred to suckling piglets (Jørgensen et al., 2025). Milk‐derived cortisol present maintained by sucking within the SOS piglets may have masked the differential response due to stress in the WN, FR, and TRANS groups.

In contrast to cortisol, the marked decrease in both T3 and T4 in all three treatment groups relative to SOS would be consistent with the hypothesis that weaning‐associated stressors can cause a suppression in circulating thyroid hormone availability. While the HPT axis is typically under strict homeostatic control, type 1 allostasis can cause concentrations of thyroid hormones to decrease. This apparent type 1 allostatic response is consistent with observations following heat stress in both swine (Macari et al., 1986) and cattle (Kahl et al., 2015). It is also consistent with observations of a NTIS response in swine following pathogenic infection (Ko et al., 2022; Pasternak et al., 2020, 2021). It is, however, worth noting that sows' milk contains T3 and T4 (Slebodziński & Cogiel, 1983), meaning piglets are deprived of this resource following weaning. As such, the observed depression in thyroid hormone could have resulted from the acute removal of this exogenous source of thyroid hormones in conjunction with delayed compensatory upregulation in endogenous production.

To better understand the source of the change in hormone concentration, we then conducted extensive gene expression analysis of the combined HPTA axis in SOS and TRANS pigs. Our results reveal a decrease in *TSHB* expression in the PIT and increased *DIO3* expression in the ROID, collectively contributing to the observed NTIS‐like response characterized by decreased circulating bioactive thyroid hormone without a compensatory increase in TSH. While there was no observable difference in circulating cortisol, expression of the ACTH receptor *MC2R* was upregulated in the TRANS group. Such upregulation may indicate increased sensitivity to ACTH, potentially counteracting the observed decrease in pituitary POMC expression.

Independent of weaning, handling and transportation create a combination of physical, nutritional, psychological, and environmental stressors for livestock. In both horses (4–15 years of age) and cattle (10–15 months of age), this combination of stressors has been associated with an increase in circulating thyroid hormone (Fazio et al., 2005, 2009). In contrast, transport has been shown to decrease serum concentrations of T3, but not T4, in adolescent pigs (Yoshioka et al., 2004). Likewise, nutritional deprivation of adolescent pigs has been shown to reduce serum T3 within 24 h, and T4 within 48 h (Buonomo & Baile, 1991). At first glance, it may therefore appear surprising that the severity of the observed allostatic response in circulating thyroid hormone was not modulated by supplemental stressors including feed restriction or transport. However, our prior work indicated that WN pigs do not consume a measurable quantity of feed in the first 9 h, despite having immediate access to feed (Turner et al., n.d.). This delay in consumption suggests that feed restriction does not cause additional stress at weaning. As regulations in the US allow for transport duration of up to 28 h without feed or water for pigs of any age (United States Code, 2025), it is possible such differences could develop when animals are transported greater distances and for longer periods of time.

The abundance of TSH is dependent on transcriptionally regulated at the level of *TSHB* (Steinfelder & Wondisford, 2009), such that the observed decrease in *TSHB* likely indicates a corresponding decrease in circulating TSH, leading to corresponding reductions in thyroid hormone production. This response mechanism would be consistent with rats, where circulating TSH is reduced in response to stress (Helmreich & Tylee, 2011). Unfortunately, circulating TSH levels were not assessed in the present study, as there is currently no validated assay available for porcine TSH, and our efforts to identify a cross‐reactive antibody have thus far been unsuccessful.

The upregulation of *DIO3* within the ROID would presumably increase the local inactivation of thyroid hormone and may therefore offer further molecular explanation for the decrease in circulating bioactive thyroid hormone concentrations. A similar response has been reported in pigs following lipopolysaccharide injection (Castro et al., 2013) and in the PRRSV‐infected fetus (Smith et al., n.d.). Increased *DIO3* action to degrade bioactive thyroid hormones would be supported by increased circulating rT3, which is observed in humans experiencing NTIS (Adler & Wartofsky, 2007). However, PRRSV‐infected porcine fetuses do not exhibit increased rT3 despite decreased circulating T3 (Ison et al., 2022). Within fetal pigs, *DIO3* is downregulated in peripheral tissues to compensate for methimazole‐induced hypothyroidism (Ison et al., 2023), but during disease challenge, there is either a decompensatory upregulation of or no change in *DIO3* expression (Ison et al., 2022). This notable difference in deiodinase expression suggests decompensatory activity in response to stress and disease induced thyroid suppression. Such activity is consistent with type 1 allostasis in which thyroid hormone metabolism is further increased in peripheral tissues like the liver and skeletal muscle (Peeters et al., 2003), despite low circulating thyroid hormone concentrations.

## 5 CONCLUSION

The present work confirms that part of the porcine stress response to weaning involves type 1 allostatic regulation of the HPTA axis, characterized by decreased thyroid hormone concentrations and altered gene expression within the ROID and PIT. The impact of stress on the HPT axis was observed in the absence of an expected stress‐induced increase in serum cortisol, suggesting increased circulating cortisol is not required for decreased thyroid hormones in the NTIS response to stress. This work gives insight into the mechanism behind the endocrine response to stress, with upregulation of *DIO3* in the ROID and downregulation of *TSHB* in the PIT suggesting a potential mechanism for the downregulation of bioactive thyroid hormones during allostasis. This observed endocrine response to weaning and associated stressors may play a role in reduced piglet performance; a cause‐and‐effect relationship between these factors has not been established.

## AUTHOR CONTRIBUTIONS

**Isabel B. Walpole:** Formal analysis; investigation; methodology. **Alyssa A. Smith:** Investigation; methodology. **Kaylyn G. Rudy:** Investigation; methodology. **Dayeon Jeon:** Investigation; methodology. **J. Scott Radcliffe:** Conceptualization; funding acquisition. **J. Alex Pasternak:** Conceptualization; funding acquisition; investigation; methodology; project administration; resources; software; supervision; visualization.

## FUNDING INFORMATION

This study was supported by the intramural research program of the U.S. Department of Agriculture, National Institute of Food and Agriculture, Agriculture and Food Research Initiative [2023‐67015‐39338]. The findings and conclusions have not been formally disseminated by the U.S. Department of Agriculture and should not be construed to represent any agency determination or policy.

## CONFLICT OF INTEREST STATEMENT

The authors declare no conflict of interest.

## ETHICS STATEMENT

The study was conducted in accordance with Purdue University Institutional Animal Care and Use Committee regulations under an approved protocol (IACUC #0123002344). Animal care and use standards were based upon the Guide for the Care and Use of Agricultural Animals in Research and Teaching (Tucker et al., 2020).

## Data Availability

The data that support the findings of this study are available from the corresponding author upon reasonable request.
